# Uric Acid: A New Perspective for Exploring the Pathological Process of Anthracycline-Induced Cardiotoxicity

**DOI:** 10.3390/cimb48010040

**Published:** 2025-12-27

**Authors:** Yifei Rao, Yu Wang, Yadi Liu, Jinjian Huang, Xueli Ding, Zhijian Lin, Bing Zhang, Xiaomeng Zhang

**Affiliations:** 1 School of Chinese Materia Medica, Beijing University of Chinese Medicine, Beijing 102488, China; ryf9696@163.com (Y.R.); wangyuxh@163.com (Y.W.); liuyadi000888@163.com (Y.L.); huangjj@bucm.edu.cn (J.H.); dingxueli202108@163.com (X.D.); linzhijian@bucm.edu.cn (Z.L.); 2 School of Traditional Chinese Medicine, Jiangxi University of Chinese Medicine, Nanchang 330004, China

**Keywords:** anthracycline-induced cardiotoxicity, hyperuricemic, pathological process

## Abstract

Anthracycline’s clinical application is often hampered by severe life-threatening cardiotoxicity, which could result in death in approximately one-third of patients. Previous studies have found that during the anthracycline-induced cardiotoxicity (AIC), uric acid (UA) levels increase abnormally. However, the role of UA in AIC remains elusive. Here, we conducted a correlation analysis between UA and cardiac damage markers (NT-pro-BNP, hs-cTnT, LDH, CRP and hs-CRP) by using the National Health and Nutrition Examination Survey database (NHANES); the results revealed that the elevated UA levels showed significant positive associations with the levels of several cardiac damage markers. Secondly, molecular docking experiments suggested potential binding interactions between UA and BNP, cTnT, CRP, and LDH. Finally, animal experiments were performed to validate this correlation we explored and further validated the effect of UA on AIC by adding or lowering UA in animal models. We observed that under high uric acid (HUA) conditions, AIC not only manifested earlier but also progressed more severely. In contrast, AIC was alleviated under UA clearance conditions. Collectively, these results suggested that HUA might be an important contributing factor in the development and progression of AIC, supporting the further investigation of UA-lowering strategies for potential prevention. This work might offer new prevention and treatment strategies for AIC.

## 1. Introduction

Cardiotoxicity and subsequent heart failure are serious complications that are commonly caused by anthracycline chemotherapy [1], which can lead to the death of about one-third of patients and result in an immense health and economic burden globally [2]. Currently, there are limited options available for the prevention and treatment of cardiotoxicity induced by anthracycline. Dexrazoxane is the only agent the Food and Drug Administration approved to prevent anthracycline-induced cardiotoxicity (AIC). The reason for this may be related to the complex pathological mechanisms of AIC that have not yet been fully understood. The mechanism of AIC involves multiple factors, including oxidative stress, inflammation, mitochondrial dysfunction, DNA damage, and triggering various cell death pathways [3,4]. However, the optimal therapeutic target remains to be further elucidated. Thus, a deep and comprehensive understanding of AIC is crucial for providing effective intervention measures. Recent clinical studies have found that anthracycline could cause abnormal increases in blood uric acid (UA) levels during anti-tumor treatment [5,6], and patients who experience cardiovascular adverse events induced after chemotherapy also have elevated blood UA levels [7]. These findings indicated that UA might play an important role in the pathological progress of AIC.

It is well established that UA is the body’s end product of purine metabolism. UA has recently been identified as an independent risk factor for cardiovascular diseases [8,9]. From the pathological perspective, increasing evidence from clinics and laboratories supports the view that UA plays a significant role in cardiovascular disease. For instance, studies have shown that elevated UA levels increase the risk of left ventricular (LV) ejection fraction reduction in patients with cardiovascular diseases [10,11,12,13]. Research has also found that elevated UA levels can promote inflammation and oxidative stress in the heart tissue, leading to cardiomyocyte hypertrophy and impaired diastolic relaxation [14]. Additionally, it has been proposed that in addition to cardiac troponin (cnTI, hs-cTnT), brain natriuretic peptides (BNP, NT-pro-BNP), C-reactive protein (CRP, hs-CRP), and lactate dehydrogenase (LDH) [15,16], UA should be used as an auxiliary biomarker for early identification of cardiovascular diseases to reduce the cost of identifying high-risk patients [17]. From the therapeutic perspective, multiple studies have demonstrated the effectiveness of lowering UA therapy in treating heart failure and improving prognosis, particularly the utilization of febuxostat, an anti-hyperuricemia agent, which can attenuate doxorubicin-induced cardiotoxicity [18,19,20]. Therefore, we speculate that UA elevation may participate in the pathological process of AIC, but further evidence is needed to confirm this potential association.

In this study, we first propose that elevated UA is involved in the pathological process of AIC and might exacerbate its progress. Firstly, we mined the National Health and Nutrition Examination Survey (NHANES) database to perform correlation analyses between UA and various cardiac damage markers (NT-pro-BNP, hs-cTnT, LDH, hs-CRP, and CRP). Subsequently, molecular docking techniques were employed to simulate the binding interactions between UA and BNP, cTnT, CRP, and LDH. Finally, hyperuricemic and UA clearance conditions were modeled in vivo to observe the effect of UA on AIC. The flowchart of the technical route is shown in the graphical abstract.

## 2. Method

### 2.1. Data Mining Based on NHANES

#### 2.1.1. Study Population

NHANES is a unique source of national data on the health and nutritional status of the US population. It periodically conducts cross-sectional surveys with nationally representative samples to collect thorough data on chronic diseases [21,22]. This survey is conducted by the National Center for Health Statistics (NCHS) at the Centers for Disease Control and Prevention (CDC) in the U.S. [23].

The NCHS Institutional Review Board approved all NHANES study protocols. Participants in NHANES provided written informed consent, and detailed descriptions were available on the official website at https://www.cdc.gov/nchs/nhanes/index.htm (accessed on 20 December 2023). This study aimed to estimate the correlation between serum UA (SUA) and cardiac damage markers (NT-pro-BNP, hs-cTnT, LDH, CRP and hs-CRP), respectively. For this analysis, Data 1–4 from different periods were extracted. Data 1 was drawn from the 1999 to 2004 cycle, which contained complete data about SUA, NT-pro-BNP, and hs-cTnT values. Data 2 was drawn from the 1999 to 2016 cycle, which contained complete data about the values of SUA and LDH. Data 3 was drawn from the 1999 to 2010 cycle, which contained complete data about the values of SUA and CRP. Data 4 was drawn from the 2015 to 2020 cycle, which contained complete data about the values of SUA and hs-CRP.

#### 2.1.2. Outcome Variable

This study’s diagnostic criteria for hyperuricemia were as follows: UA level higher than 420 μmol/L in men or women [24], and defined cardiac toxicity as NT-pro-BNP ≥ 450 pg/mL [25,26], hs-cTnT ≥ 14 ng/L [27,28], LDH ≥ 200 U/L [29,30], CRP ≥ 1.0 mg/dL [31] and hs-CRP ≥ 3.0 mg/dL [32].

#### 2.1.3. Covariates

This investigation included covariates that may impact the relationship between SUA and NT-pro-BNP, hs-cTnT, LDH, hs-CRP, and CRP. Demographic parameters included age, gender, race, and BMI. Health risk factors included hyperlipidemia, hypertension, and diabetes mellitus.

#### 2.1.4. Statistical Analyses

For the data analysis of NHANES, data extraction and analysis were performed using R version 4.2.1 through the “nhanesR” package. Continuous variables were shown as survey-weighted means with standard error (SE), whereas categorical variables were expressed as percentages (%). *p* < 0.05 was considered statistically significant. We performed multivariate logistic regression models for primary analyses to analyze the association between SUA and NT-pro-BNP, hs-cTnT, LDH, CRP, and hs-CRP. Odds ratio (OR) with 95% confidence interval (CI) was used to determine the degree of association. We did not adjust for any covariate in Model 1, and adjusted for age (years) and race (Mexican American, other Hispanic, non-Hispanic white, non-Hispanic black, other race) in Model 2. Model 3, the fully adjusted model, was further adjusted for BMI, hyperlipidemia (yes/no) and hypertension (yes/no), and diabetes mellitus (yes/no). Subgroup analysis was carried out to investigate the relationship between NT-pro-BNP, hs-cTnT, LDH, CRP and hs-CRP and SUA in different subgroups. We further carried out stratified analysis though dividing into quartiles based on the distribution level of SUA. Additionally, we investigated the influence of SUA levels on the relationship between cardiac damage markers (NT-pro-BNP, hs-cTnT, LDH, CRP and hs-CRP), respectively.

### 2.2. Molecular Docking Experiments

Molecular docking experiments were conducted using AutoDock tools 1.5.7 and Vina (i.e., AutoDock Vina) 1.2.0. The specific process was as follows:

(1) Preparation of the protein receptor PDBQT files: Retrieve the structures of the protein from the Protein Data Bank (PDB, http://www.rcsb.org/), and then import it into the Pymol 3.0.3 software for pre-treating (including the removal of water molecules, salt ions, and other small molecules from the protein results). Subsequently, in the AutoDock tools 1.5.7 software, the non-polar hydrogen atoms of protein were merged into the corresponding carbon atoms, and the missing hydrogen atoms and Kollman partial charges were added to the protein. The protein files were then saved in PDBQT format.
(2) Preparation of 3D structures of small molecules: The 3D structures were downloaded from the PubChem database (https://pubchem.ncbi.nlm.nih.gov/) and modified by the addition of hydrogens and protonation in AutoDock tools; they were finally saved as PDBQT format.
(3) Establishment of mating pockets: The PDBQT files of proteins were utilized for the construction of mating pockets. The appropriate size was set for the mating box to keep the protein totally covered by the box for blind docking, and output files were in configuration (config) format.
(4) Docking and visualization: Vina software was applied for rigid docking and calculated the binding affinity based on the proteins and small molecules. The highest-scoring docked conformations from the molecular docking were the output. The interactions of docking models were obtained by Protein–Ligand Interaction Profiler (PLIP, https://plip-tool.biotec.tu-dresden.de/plip-web/plip/, (accessed on 30 December 2023)) and visualized by Pymol.

### 2.3. Animal Experiments

#### 2.3.1. Experimental Design

Male SD rats (220 ± 10 g) were purchased from Beijing Si Bei Fu Biotechnology (Certificate SCXK-2020-0033) and acclimatized for 3 days in the Beijing University of Chinese Medicine’s animal facility. The rat models of cardiotoxicity were established through intraperitoneal injection of doxorubicin (DOX) at a dose of 3.5 mg/kg every three days. To investigate the effects of different UA levels on cardiac toxicity in rats, we created the AIC model under simulated hyperuricemic (HUA) conditions (HUA+AIC group), as well as the AIC model under UA clearance conditions (AIC+Allopurinol/Benzbromarone group). The HUA+AIC group was induced into a hyperuricemic state by gavage administering a combination of potassium oxonate (PO, 750 mg/kg) and yeast extract (YE, 10 g/kg) based on the AIC model. The AIC+Allopurinol and AIC+Benzbromarone groups were gavage administered with allopurinol (20 mg/kg) and benzbromarone (20 mg/kg), respectively, during the construction of the AIC model, to establish a state of UA clearance. The control group received only saline (0.9% NaCl). Cardiac function was assessed by echocardiography (ECHO) and electrocardiogram (ECG). Subsequently, UA and cardiac injury markers (cTnI, BNP, and NT-proBNP) were detected by reagent kits. Rats were euthanized at the endpoint, and their heart tissues were removed for histological and indicator detection. All the animal experiments were conducted according to proven guidelines specified by the animal ethics committee of the Beijing University of Chinese Medicine (Beijing, China; No. BUCM-4-2023030904-1066).

#### 2.3.2. Cardiac Function Assessment

Echocardiography was performed with the Vevo 2100 echocardiography system (VisualSonics, Toronto, ON, Canada). Rats were anesthetized with 1.5% sodium pentobarbital through intraperitoneal injection. Next, the rats’ chests were shaved, and hair removal gel was applied to minimize resistance to ultrasonic beam transmission. Rats were laid supine on a warm platform, and a series of M-mode images at the level of papillary muscles was obtained. Notably, ejection fraction (EF), fractional shortening (FS), left ventricular anterior wall in systolic (LVAW; s), left ventricle internal diameter in systolic (LVID; s), left ventricular posterior wall in systolic (LVPW; s), and left ventricle volume in systolic (LV Lol; s) were measured based on three consecutive cardiac cycles.

Subsequently, the anesthetized rats continued to receive electrocardiogram (ECG) measurements. The rats were fixed in a supine position, and the ECG was recorded through a BL-420S biological function experiment system (Chengdu Taimeng Software Co., Ltd., Chengdu, China) to inspect the cardiac function.

#### 2.3.3. Detection of UA Levels and Cardiac Injury Biomarkers

Blood samples were taken from the abdominal aorta and centrifuged at 3500 rpm for 10 min after being left to stand. The frozen heart tissue (50 mg) was homogenized in a 400 μL phosphate-buffered saline. The lysate was then centrifuged at 5000× *g* for 15 min at 4 °C. Supernatants were collected to assay serum/tissue UA and cardiac injury biomarkers levels. UA levels were measured using an assay kit (Zhongsheng Beikong Biotechnology Co., Ltd., Beijing, China), following the manufacturer’s instructions. The levels of cTnI, BNP, and NT-proBNP were measured using enzyme-linked immunosorbent assay (ELISA) detection kits, as directed by the manufacturer (Jiangsu Meimian Industrial Co., Ltd., Jiangsu, China).

#### 2.3.4. Histological Evaluation

Paraformaldehyde (4%) was added to the myocardial tissue samples and left overnight. The tissues were then embedded, sectioned (3 μm), and stained with hematoxylin and eosin (H&E), as well as Masson trichrome. The staining was performed to identify histological alterations in the myocardial structure. The histological sections were imaged or fully scanned using a microscope (Leica, Wetzlar, Germany) at 200× *g* and 400× *g* magnification. Following that, to obtain the results of Masson staining, the percentage of collagen fiber areas was calculated using the Image-Pro Plus 6.0 software.

#### 2.3.5. Transmission Electron Microscopy

The integrity and the morphological change in mitochondria were observed by transmission electronic microscopy (TEM). Samples of myocardial tissue were fixed by the pre-cooled 2.5% glutaraldehyde; then, after a series of ethanol dehydration steps, embedding, polymerization and sectioning were performed; subsequently, the 60–80 nm thick sections (Leica EM UC7, Wetzlar, Germany) were stained by 2% uranyl acetate and 2.6% lead citrate, respectively. Finally, images were acquired and visualized by electron microscopy (HITACHI HT 7800, Dongjing, Japan).

### 2.4. Statistical Analysis

Data were analyzed with IBM SPASS Statistics 20 (IBM Corp, Armonk, NY, USA). All values are presented as the mean ± standard deviation (SD). For the analysis of experimental data, the normality of data within each group was assessed using the Shapiro–Wilk test, and the homogeneity of variances was evaluated using Levene’s test. For data with normal distribution, one-way ANOVA was performed. In cases of homogeneous variances, post hoc comparisons were conducted using the LSD test; for data with heterogeneous variances, the Dunnett-t test was applied. For data that did not follow normal distribution, the nonparametric Kruskal–Wallis H test was used, followed by Dunn’s correction for post hoc comparisons. Statistical significance was set at *p* < 0.05 and *p* < 0.01. All figures were produced using GraphPad Prism 9.0.0 (GraphPad Software Inc., San Diego, CA, USA).

## 3. Result

### 3.1. NHANES Data Mining Discovered the Positive Correlation Between UA and NT-pro-BNP, hs-cTnT, LDH, hs-CRP, and CRP

#### 3.1.1. Baseline Characteristics of Participants

Extracting NT-pro-BNP and UA simultaneously in the database of 18,024 participants meeting the criteria, and the basic elements are listed in Supplementary Table S1. The average age was 42.24 years, and the gender split was 48.50% male to 51.50% female. The 18,024 participants represented the 202.9 million non-institutionalized civilian population of the United States. Extracting hs-cTnT and UA simultaneously, 18,022 participants were involved, and the basic characteristics are listed in Supplementary Table S2. The average age was 42.25 years, and the experiment had a gender split of 48.50% men to 51.50% women. The 18,022 participants represented the 202.8 million non-institutionalized civilian population of the United States. Extracting LDH and UA simultaneously, 53,613 participants meeting the criteria were included, and the basic characteristics are listed in Supplementary Table S3. The average age was 43.07 years, and the gender split was 48.50% male to 51.50% female. The 53,613 participants represented the 232.11 million non-institutionalized civilian population of the United States. Extracting hs-CRP and UA simultaneously, 21,554 participants meeting the criteria were included, and the basic characteristics are listed in Supplementary Table S4. The average age was 44.51 years, and the gender split was 48.63% male to 51.37% female. The 21,554 participants represented the 253.4 million non-institutionalized civilian population of the United States. Extracting CRP and UA simultaneously, 39,252 participants meeting the criteria were included, and the basic characteristics are listed in Supplementary Table S5. The average age was 42.27 years, and the gender split was 48.61% male to 51.39% female. The 39,252 participants represented the 224.1 million non-institutionalized civilian population of the United States. Hyperuricemia in patients was different with statistical significance of age, height, weight, BMI, waist circumference, gender, race/ethnicity, hyperlipidemia, hypertension, and diabetes mellitus (DM) (all *p* < 0.01). Notably, the analyzed population did not contain information regarding chemotherapy exposure, cancer diagnosis, or anthracycline treatment. Therefore, this analysis only provided a preliminary assessment of the association between UA and cardiac damage markers within the general U.S. population.

#### 3.1.2. Associations Between SUA and NT-pro-BNP, hs-cTnT, LDH, CRP and hs-CRP

When using correlation analysis, there was a positive correlation between SUA and NT-pro-BNP, hs-cTnT, LDH, CRP, and hs-CRP. The result of univariate analysis of logistic regression models is shown in Table 1. SUA was significantly associated with an increased risk of NT-pro-BNP (OR = 2.62, *p* = 2.94 × 10^−12^), hs-cTnT (OR = 3.39, *p* < 2 × 10^−16^), LDH (OR = 1.69, *p* = 2.26 × 10^−10^), CRP (OR = 1.42, *p* = 4.59 × 10^−8^), and hs-CRP (OR = 1.84, *p* = 5.97 × 10^−14^) in the crude model (Model 1). After multivariable adjustment, the results remained robust and statistically significant, adjusting for age, gender, and race (Model 2) (OR > 1, *p* < 0.001) and further adjusting for BMI, hyperlipidemia, hypertension, and diabetes mellitus (Model 3) (OR > 1, *p* < 0.05).

#### 3.1.3. Subgroup Analysis

As shown in Table 2, subgroup analysis results revealed that a significant association of HUA with the level of NT-pro-BNP, hs-cTnT, LDH, CRP, and hs-CRP was observed in the HUA population for the stratified subgroup when dividing into quartiles based on the distribution level of SUA. In Model 3, which adjusts for all variables, the level of NT-pro-BNP (Q1 vs. Q3: OR = 2.60, 95% CI = 1.19–5.65, *p* = 0.022; Q1 vs. Q4: OR = 3.97, 95% CI = 1.89–8.31, *p* = 0.001), hs-cTnT (Q1 vs. Q4: OR = 3.29, 95% CI = 1.92–5.63, *p* < 0.001), LDH (Q1 vs. Q3: OR = 1.78, 95% CI = 1.08–2.91, *p* = 0.024; Q1 vs. Q4: OR = 2.16, 95% CI = 1.28–3.64, *p* = 0.005), CRP (Q1 vs. Q4: OR = 1.65, 95% CI = 1.09–2.50, *p* = 0.020), and hs-CRP (Q1 vs. Q4: OR = 1.66, 95% CI = 1.02–2.7, *p* = 0.048) were independently associated with the HUA, indicating that HUA is associated with the increased level of NT-pro-BNP, hs-cTnT, LDH, CRP, and hs-CRP levels. However, it is important to note that the NHANES population was not specifically identified for AIC, and therefore, the observed associations cannot be directly attributed to anthracycline exposure. The NHANES data analysis could only demonstrate an association between UA levels and cardiac damage markers in the general population; the direct association analysis between UA and AIC required further validation in anthracycline-treated cohorts.

### 3.2. The Molecular Docking Results of UA and Cardiac Damage Markers (BNP, cTnT, LDH, and CRP)

**Figure 1:**
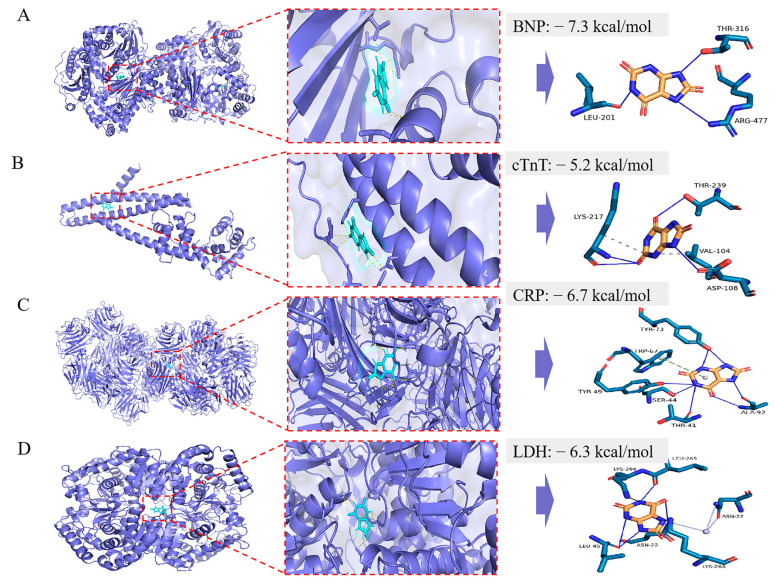
The molecular docking model of UA with BNP (**A**), cTnT (**B**), CRP (**C**), and LDH (**D**), respectively. In the binding model, hydrophobic interactions are indicated by gray dashed lines, hydrogen bonds by navy blue solid lines, salt bridges by yellow dashed lines, π-stacking (parallel) by bright green dashed lines, π-stacking by dark green dashed lines, and π-cation interactions by orange dashed lines.

The molecular docking was performed using AutoDock Vina. The docking energy of UA with BNP, cTnT, LDH, and CRP was then calculated (Table 3). The lower the docking energy of the complex, the more stable the receptor–ligand binding conformation. Then, docking diagrams (Figure 1) were further generated to identify the binding sites of the UA. For BNP, as shown in Figure 1A, UA could form hydrogen bonds with LEU-201 (length = 2.59), THR-316 (length = 1.93), and ARG-477 (length = 2.70). For cTnT, as shown in Figure 1B, UA could form hydrophobic interactions with VAL-104 (length = 3.48) and LYS-217 (length = 3.87). Hydrogen bonds with ASP-108 (length = 2.50), LYS-217 (length = 2.02, 2.56), and THR-239 (length = 3.03). For CRP, as shown in Figure 1C, UA could form hydrogen bonds with THR-41 (length = 3.28), SER-44 (length = 2.69), TYR-49 (length = 3.41), TYR-73 (length = 2.41, 3.02, 2.90), ALA-92 (length = 2.45, 2.51), and a π-stacking with TRP-67 (length = 5.42). For LDH, as shown in Figure 1D, UA could form hydrogen bonds with ASN-22 (length = 3.38), LEU-45 (length = 3.25, 2.62), LEU-265 (length = 2.37), and LYS-266 (length = 2.83, 2.89), as well as water bridges with ASN-22 (length = 2.65, 3.93).

### 3.3. Premature Occurrence and Exacerbates of AIC in Rats Under HUA State

**Figure 2:**
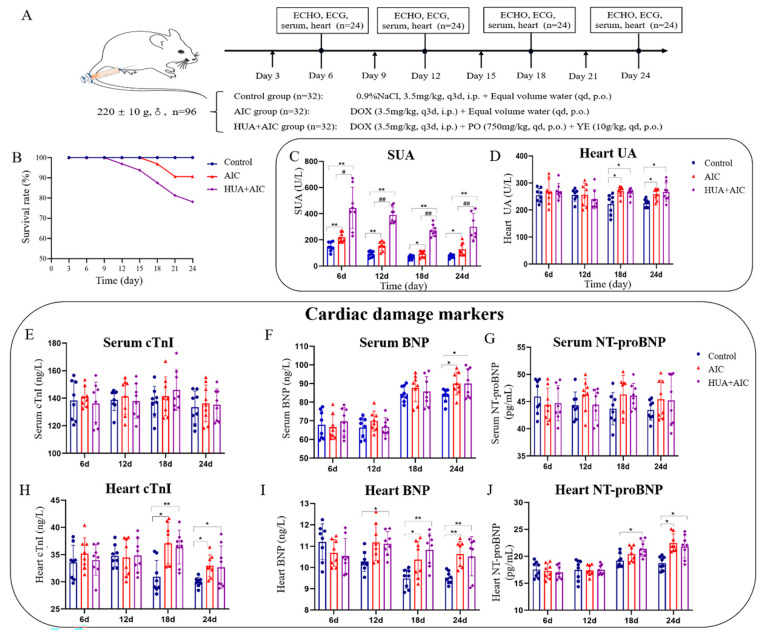
Hyperuricemia is involved in the development and progression of anthracycline-induced-cardiotoxicity. (**A**) Flowchart of the experiment. (**B**) The survival rate of the animal. (**C**) Quantitative analysis of uric acid level in serum on days 6, 12, 18, and 24. (**D**) Quantitative analysis of uric acid level in the heart tissue on days 6, 12, 18, and 24. (**E**–**G**) Quantitative analysis of cardiac troponin I (cTnI), brain natriuretic peptide (BNP), and n-terminal pro-brain natriuretic peptide (NT-proBNP) in serum on days 6, 12, 18, and 24. (**H**–**J**) Quantitative analysis of cardiac troponin I (cTnI), brain natriuretic peptide (BNP), and n-terminal pro-brain natriuretic peptide (NT-proBNP) in heart tissue on days 6, 12, 18, and 24. Compared with the control group, * *p* < 0.05, ** *p* < 0.01. Compared with the anthracycline-induced cardiotoxicity (AIC) group, ^#^ *p* < 0.05, ^##^ *p* < 0.01.

During the experimental period, we monitored SUA and heart UA levels every 6 days (every increase of 7 mg/kg in DOX cumulative dose) (Figure 2A). Results revealed that the SUA level in the AIC group was significantly higher than the control group from day 6 to the end of the experiment (Figure 2C). The heart UA level increased dramatically in the AIC group compared to the control group on day 18 (DOX cumulative dose ≥ 21 mg/kg), which is the day on which the AIC model had already been established, and the rat survival rate in the AIC group decreased to 90.625% (Figure 2B,D). These results suggest that abnormally elevated UA levels in the heart might be one of the reasons exacerbating AIC and leading to rat death.

To confirm the role of UA in AIC, we administered AIC rats with high levels of UA. Results showed that compared to the control group, the SUA levels in the HUA+AIC group and the AIC group increased significantly throughout the whole experimental period, and the UA levels in the heart began to increase from day 18 (DOX cumulative dose ≥ 21 mg/kg). Notably, only SUA showed significant differences between the HUA+AIC and AIC groups, and the UA levels in the heart of the two groups tended to be consistent. At the same time, compared to the AIC group, the general condition and cardiac function of the HUA+AIC group rats showed significant deterioration. For example, on day 12 of the experiment (DOX cumulative dose of 14 mg/kg), the HUA+AIC group rats began to die, while the AIC group rats had not yet died. At the end of the experiment, the survival rate of the HUA+AIC group rats was only 78.13%, which was significantly lower than that of the AIC group rats (Figure 2B).

Cardiac injury markers (cTnI, BNP, and NT proBNP) were used to assess the status of cardiac injury. As shown in Figure 2E–J, the BNP and NT-proBNP levels in the heart in the HUA+AIC group were elevated earlier compared to the AIC group, which suggests an earlier cardiac injury in the HUA+AIC group as compared to the AIC group; this is consistent with the results of the cardiac function assessment. There were insignificant changes in the serum cardiac injury markers among experimental groups, possibly due to the insufficient sensitivity of serum heart damage markers.

**Figure 3:**
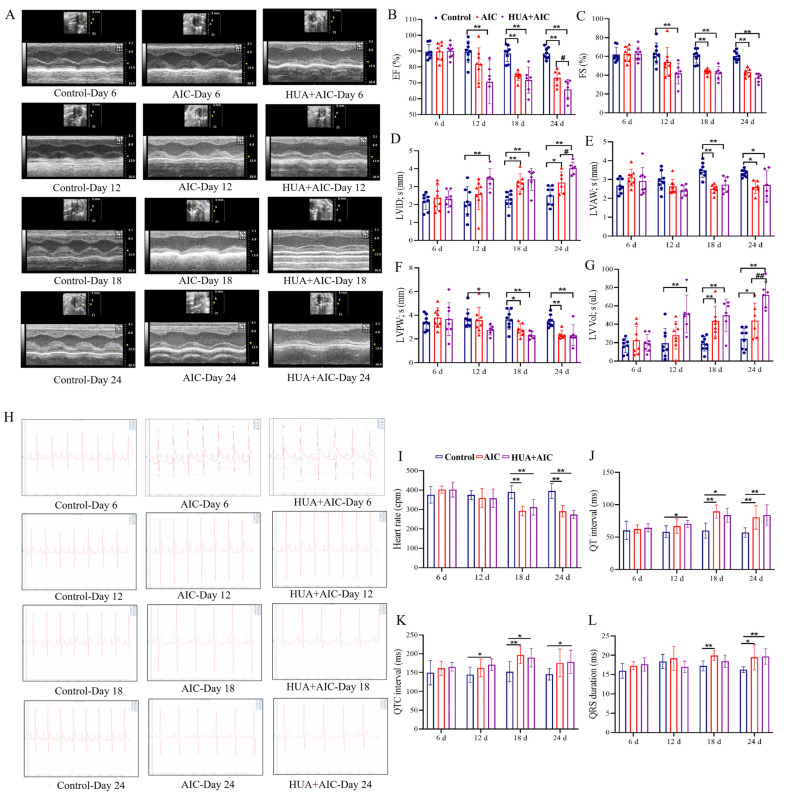
Cardiac dysfunction was worsened in AIC rats under hyperuricemic conditions. (**A**) Representative images of echocardiographic measurements at different times in each group. (**B**–**G**) Quantitative analysis of ejection fraction (EF), fractional shortening (FS), left ventricular anterior wall in systolic (LVAW; s), left ventricle internal diameter in systolic (LVID; s), left ventricular posterior wall in systole (LVPW; s), and left ventricle volume in systolic (LV Lol; s). (**H**) Representative images of electrocardiogram measurements at different times in each group. (**I**–**L**) Quantitative analysis of heart rate, PR interval, QT interval, QTC interval, and QRS duration. Compared with the control group, * *p* < 0.05, ** *p* < 0.01. Compared with the anthracycline-induced cardiotoxicity (AIC) group, ^#^ *p* < 0.05, ^##^ *p* < 0.01.

Echocardiography (ECHO) and electrocardiogram (ECG) were applied to assess the degree of cardiac injury from the aspect of cardiac function. From the ECHO result in Figure 3A–G, on day 12, the HUA+AIC group rats began to exhibit significant cardiac dysfunction, characterized by significant decreases in EF, FS, and LVPW; s, as well as increases in LVID; s and LV Lol; s, while the AIC group of rats did not yet show cardiac dysfunction. At the end of the experiment, HUA+AIC group rats exhibited significantly worsened cardiac function compared to the AIC group, manifested as a decrease in EF value (*p* < 0.05, 95% CI [1.34, 13.54]), an increase in LVID; s (*p* < 0.05, 95% CI [−1.41, −0.19]), and an increase in LV Lol; s (*p* < 0.01, 95% CI [9.97, 46.18]). Furthermore, similarly, on day 12, ECG showed that the HUA+AIC group exhibited an earlier occurrence of prolongation in QT and QTC intervals compared to the AIC group (Figure 3H–L), which indicates that the HUA+AIC group rats developed arrhythmias at an earlier stage than AIC group rats.

**Figure 4:**
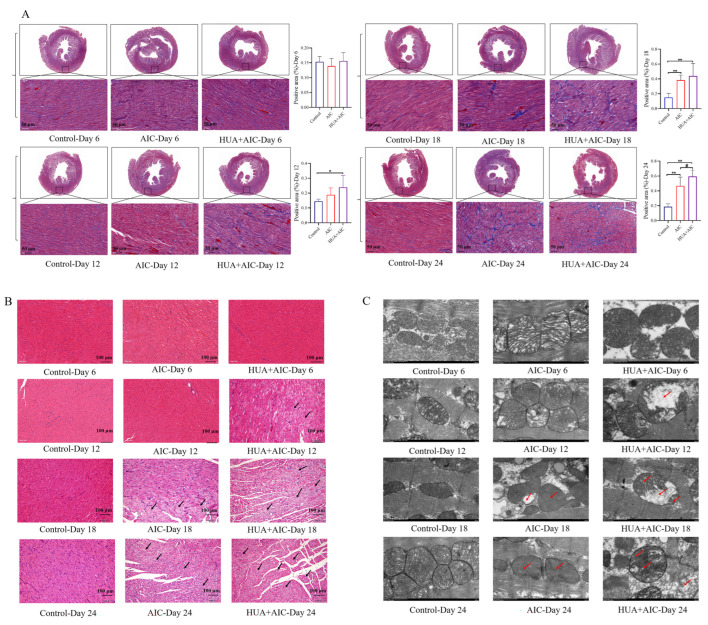
Hyperuricemia might become a risk factor worthy of consideration in anthracycline-induced cardiotoxicity. (**A**) The percentage of collagen fibers quantified by Masson staining from heart sections of each group and the positive area. Images were captured at 400× *g* magnification. (**B**) Representative hematoxylin and eosin staining images in each group were captured at 200× *g* magnification. (**C**) Representative images of transmission electronic microscopy in each group were captured at ×12,000 magnification. Compared with the control group, * *p* < 0.05, ** *p* < 0.01. Compared with the AIC group, ^#^ *p* < 0.05. The area pointed to by the arrow was the clearly visible lesion site.

In addition, histopathologic change in the heart was observed. Masson staining quantitative analysis was performed to evaluate the deposition of collagen fibers in the heart. The results demonstrated that compared to the control group, the deposition of cardiac collagen fibers in the HUA+AIC and AIC groups increased from day 12. At the end of the experiment, compared with the AIC group, the HUA+AIC group showed a significant increase in collagen fiber deposition (*p* < 0.05, 95% CI [0.01, 0.25]) (Figure 4A). HE staining showed a slight inflammatory infiltrate in the HUA+AIC group from day 12. On day 18th, both HUA+AIC and AIC groups showed significant myocardial pathologic damage, with myocardial fiber breakage and obvious inflammatory infiltration. At the end of the experiment, the HUA+AIC group showed more severe myocardial fiber breaks and inflammatory infiltration than the AIC groups (Figure 4B). Then, we observed the morphological changes in mitochondria under TEM (Figure 4C); on day 12, the HUA+AIC group showed mitochondrial swelling, vacuolation, membrane rupture, and cristae reduction. On day 18, mitochondrial swelling and fracture, abnormal shape, partial membrane rupture, cristae reduction, and vacuolization were observed in the HUA+AIC group and AIC group. By the end of the experiment, mitochondrial damage in the HUA+AIC group was significantly worse than that in the AIC group, characterized by the disappearance of cristae and the fading of the intra-membrane matrix.

These results show that SUA and heart UA levels were continuously elevated in AIC, suggesting that UA might be involved in constructing AIC. Furthermore, under HUA conditions, AIC rats exhibited decreased overall survival and aggravated cardiac dysfunction. These findings suggested that HUA might be a risk factor worthy of consideration in AIC, indicating that controlling UA levels could be a potential effective measure for AIC intervention.

### 3.4. Alleviation of AIC in Rats with a Lowering UA Agent

**Figure 5:**
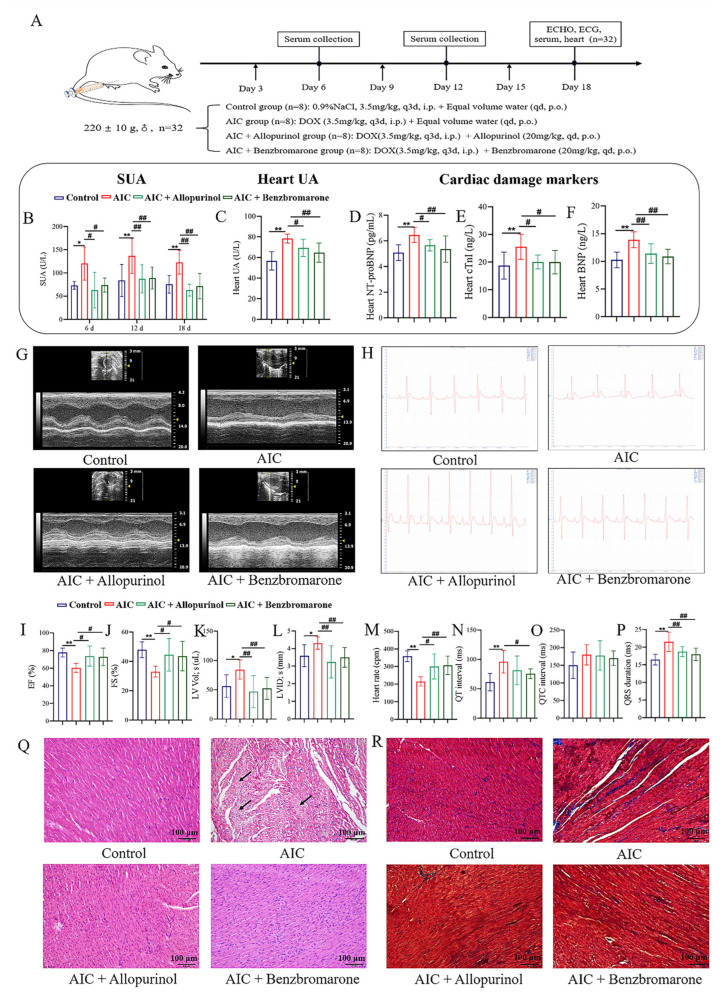
Uric acid lowering intervention for ameliorated anthracyclines-induced cardiotoxicity. (**A**) Flowchart of the experiment. (**B**) Quantitative analysis of uric acid level in serum in each group. (**C**) Quantitative analysis of uric acid level in heart tissue in each group. (**D**–**F**) Quantitative analysis of n-terminal pro-brain natriuretic peptide (NT-proBNP), cardiac troponin I (cTnI), and brain natriuretic peptide (BNP). (**G**) Representative images of echocardiographic measurements in each group. (**I**–**L**) Quantitative analysis of ejection fraction (EF), fractional shortening (FS), left ventricle volume in systolic (LV Lol; s), and left ventricle internal diameter in systolic (LVID; s). (**H**) Representative images of electrocardiogram measurements at different times in each group. (**M**–**P**) Quantitative analysis of heart rate, QT interval, QTC interval, and QRS duration. (**Q**) Representative hematoxylin and eosin staining images in each group were captured at 200× *g* magnification. (**R**) Representative images of Masson staining in each group were captured at 200× *g* magnification. Compared with the control group, * *p* < 0.05, ** *p* < 0.01. Compared with the AIC group, ^#^ *p* < 0.05, ^##^ *p* < 0.01. The area pointed to by the arrow was the clearly visible lesion site.

To explore whether controlling UA could alleviate AIC, AIC rats administrated with allopurinol and benzbromarone, two lowering-UA agents that inhibit UA production or promote UA excretion, respectively, were employed. Drug treatment is indicated in the schematic plan (Figure 5A). Figure 5B,C show that SUA and heart UA levels in the AIC group were significantly higher than in the control group during the experimental time. Compared to the AIC group, the SUA and heart UA levels in the AIC+allopurinol group and the AIC+benzbromarone group were significantly reduced.

Subsequently, we detected the cardiotoxicity biomarkers, including cTnI, BNP, and NT-proBNP, and assessed cardiac function with echocardiography and electrocardiogram to evaluate cardiac injury in each group. From the results of Figure 5D–F, increased levels of cTnI, BNP, and NT-proBNP in the heart tissue in the AIC group were observed compared to the control group. In contrast, those cardiac biomarkers were markedly reduced after allopurinol and benzbromarone treatment. From the results of Figure 5G,I–L, ECHO showed that the cardiac function of the AIC group was impaired, characterized by significant decreases in EF and FS compared to the control group, while LV Lol; s and LVID; s increased significantly. However, improvement in cardiac function was observed in AIC rats treated with allopurinol or benzbromarone, characterized by increases in EF and FS, while LV Lol; s and LVID; s decreased.

From the ECG results in Figure 5H,M–P, compared to the control group, the heart rate of the AIC group decreased significantly, and its QT interval and QRS interval were prolonged. However, after the intervention of lowering UA, the ECG of the AIC rats showed significant improvements, as reflected in enhancing heart rate and the shortening of QRS duration in the AIC+allopurinol group; the significant improvement in heart rate and the significantly shortened QT interval and QRS duration in the AIC+benzbromarone group is also observed.

Furthermore, we applied H&E staining and Masson staining to evaluate the structural changes in the heart tissue in each group of rats. As shown in Figure 5Q, the myocardial structure in the control group was clear, the myocardial cell tissue was orderly, and no inflammatory cell infiltration was observed. In the AIC group, the myocardial arrangement was irregular, myocardial fibers were broken, and inflammatory cell infiltration was prominent. However, after intervention with allopurinol and benzbromarone, the myocardial structure and inflammatory infiltration in the AIC rats were significantly improved. As shown in Figure 5R, compared to the control group, the collagen fibers in the interstitial tissue of the AIC group were enhanced considerably and distributed in a disordered manner. Compared to the AIC group, the collagen fibers in the interstitial tissue of the AIC+allopurinol and AIC+benzbromarone groups were lesser.

The above results suggest that reducing UA levels could effectively alleviate AIC, reflecting the essential pathological role of HUA in promoting the development and occurrence of AIC and highlighting the prospect of lowering UA in the prevention and treatment of AIC.

## 4. Discussion

Yesterday’s cancer survivors are rapidly becoming today’s heart failure patients. Nowadays, chemotherapy-induced cardiotoxicity is bringing a new set of healthcare challenges. Anthracyclines are the cornerstone of multiple chemotherapy regimens for various cancers but could lead to various cardiac injuries from anthracycline exposure, subclinical myocardial cellular dysfunction, to heart failure [33]. Previous studies have classed AIC into acute, early-onset chronic, and late-onset chronic cardiotoxicity. Acute cardiotoxicity presents as arrhythmias, elevated BNP and cTn levels, and reversible cardiac dysfunction. Its histopathological changes manifest as myocyte damage and inflammatory infiltrates are usually reversible after therapy discontinuation. Chronic cardiotoxicity presents as pathological myocardial remodeling typified by cardiomyocyte hypertrophy and increased fibrosis, leading to irreversible functional cardiac decline [34]. However, the latest research has proposed that AIC is a continuous phenomenon that starts from myocardial cell injury, followed by left ventricular ejection fraction, and progressively leads to symptomatic heart failure [35]. Although the mechanisms of AIC have been studied extensively, including oxidative stress, inflammatory response, mitochondrial injury, endoplasmic reticulum (ER) stress, calcium (Ca^2+^) dyshomeostasis, apoptosis, fibrosis, and dysregulation of autophagy [36], the exact mechanism of anthracycline cardiotoxicity is unclear. Clinically, dexrazoxane remains the only drug that works as a cardioprotective agent against AIC, but it inevitably induces adverse effects, such as myelosuppression and secondary malignancies [37]. Hence, efforts are still underway to clarify molecular mechanisms and pinpoint more effective pharmacological treatment of AIC.

An adequate observation of the AIC formation process is crucial for understanding its pathogenesis and taking preventive measures. In the present study, we observed the progression of AIC, including cardiac function and cardiac injury marker expressions, every 6 days (every increase of 7 mg/kg in DOX cumulative dose) to investigate the dynamic pathological development of AIC. Among the numerous detection indicators, we found it interesting that SUA levels increased throughout the entire AIC process, while UA levels in the cardiac tissue were only elevated after establishing the AIC model. This indicates the involvement of UA in the whole progression of AIC. Meanwhile, combined with the observation in animal experiments that AIC rats under HUA conditions exhibited reduced survival and more severe cardiac dysfunction, while heart UA levels did not increase significantly in parallel with SUA levels, this paradox indicates that the exacerbating effect of HUA on AIC might not be mediated by the direct accumulation of UA in the myocardium. Instead, it is likely mediated by systemic pathological states induced by HUA, such as systemic inflammation, oxidative stress, and endothelial dysfunction.

UA, the end product of purine metabolism [38], is an important antioxidant at physiological concentrations, which is well known. Still, at abnormally elevated concentrations, it can become a risk factor for hyperuricemia and gout [39]. Recently, elevated UA levels have also been recognized as an independent marker for many cardiovascular diseases, such as atherosclerosis, hypertension, atrial fibrillation (AF), and heart failure (HF) [40,41,42,43]. Notably, increased UA levels have been observed in patients receiving anthracycline chemotherapy and the cardiac tissues of rats that received DOX [44]. Meanwhile, clinical studies have also observed some indicators of AIC cardiac injury, such as elevated levels of cnTI, hs-cTnT, BNP, NT-pro-BNP, CRP, and LDH, which often occur together with increased UA level [45]. Specifically, elevated levels of cTnT and UA co-occurred in acute myocardial infarction patients, and combining UA with cTnT improved the diagnostic accuracy offered by cTnT alone. This benefit seemed to be pronounced, especially in early presenters and patients with initially undetectable cTnT [46]. Another research also showed that the myocardial performance index, which was used to assess LV dysfunction and LV hypertrophy, was independently associated with UA and hs-cTnT [47]. Meanwhile, a study regarding acute coronary syndrome reported that UA and NT-proBNP were positively correlated with the severity of coronary artery [48]. High plasma UA level, partly secreted from the failing heart, is a prognostic predictor independent of BNP in patients with congestive heart failure. Monitoring a combination of BNP and UA may be useful for the management of patients with congestive heart failure [49]. Moreover, CRP and UA are both potentially good prognostic markers in heart failure and can also be used as adjunctive parameters in the early diagnosis and follow-up of right cardiac disorders [50,51]. Several studies have shown the increased CRP expression may be linked to the adverse effects of UA on the vasculature. For example, UA could up-regulate CRP expression in cultured human vascular cells, that may provide further direct evidence for both proinflammatory and proatherogenic effects of UA [52]. In addition, a combination of hs-CRP and UA was considered useful for predicting the development of heart failure in acute myocardial infarction patients [53]. Overall, studies suggest that the higher the level of UA, the higher the risk of cardiac injury markers, indicating that UA may have a significant and independent adverse effect on AIC. However, more comprehensive and systematic studies are needed to clarify the associations between UA and AIC. Thus, NHANES, a nationally representative cross-sectional survey, was employed in this study. We found that there is a positive correlation between UA levels and five cardiac injury markers (NT-pro-BNP, hs-cTnT, LDH, hs-CRP, and CRP), be it adjusting demographic parameters and health risk factors or using multiple logistic regression models. Molecular docking techniques were employed to simulate the binding interactions between UA and BNP, cTnT, LDH, and CRP, respectively. Although these analyses were not derived directly from AIC patients, given that cardiac injury markers were commonly used to identify potential AIC, these results suggested that UA might have similar implications in AIC.

Subsequently, we conducted animal experiments to verify the role of UA in the progression of AIC. AIC rats administered with exogenous high-concentration UA and AIC rats administered with UA-lowering drugs were used. Our finding suggested that the elevated UA level speeds up the occurrence and development of AIC while reducing UA levels can alleviate it, confirming that HUA might become a risk factor worthy of consideration in AIC. However, its correlated mechanism is unclear. Recently, UA has been considered the biomarker of oxidative damage since HUA could aggravate inflammation, cell apoptosis, endothelial dysfunction, and abnormal energy metabolism in cardiomyopathy by promoting oxidative stress, which is one of the essential mechanisms of AIC [2,54,55]. Concretely, HUA could induce mitochondrial ROS production and activate inflammation by directly inhibiting the mitochondrial protein UCP2 [56]. HUA could also aggravate myocardial injury through the ROS/NLRP3 pyroptosis pathway [57]. Fibrosis of atria induced by HUA may relate to the increase in ROS which is activated by the TGF-β1 signaling pathway-induced fibroblast differentiation [58,59]. At the same time, some researchers think the close correlation between UA and AIC is based on a complex pathophysiological mechanism that probably involves the extensive activation of the enzyme xanthine-oxidase (XO), which is responsible for UA production [60]. A recent study suggested that XO is activated in congestive heart failure, and the increase in XO action is also assumed to be the trigger that leads to increased cytokine levels, endothelial dysfunction, apoptosis, and energetic alterations in myocardial cells [61]. Further in-depth experimental research is needed to uncover its mysterious veil.

## 5. Conclusions

This study uncovered that HUA might be a risk factor in accelerating AIC occurrence and progress, and early intervention in lowering HUA levels may prevent AIC deterioration. We provided new insight into the relationship between HUA and AIC, and offered new prevention and treatment strategies for AIC. Further studies are required to clarify the detailed mechanisms of the interactions between HUA and AIC.

## Figures and Tables

**Table 1:** Association between SUA and NT-pro-BNP, hs-cTnT, LDH, CRP, and hs-CRP.

	Model 1		Model 2		Model 3	
	OR	*p* value	OR	*p* value	OR	*p* value
NT-pro-BNP	2.62	2.94 × 10^−12^ ***	1.99	1.59 × 10^−7^ ***	1.93	2.93 × 10^−6^ ***
hs-cTnT	3.39	<2 × 10^−16^ ***	2.40	9.60 × 10^−11^ ***	2.27	7.13 × 10^−9^ ***
LDH	1.69	2.26 × 10^−10^ **	1.52	2.37 × 10^−6^ ***	1.32	0.003 **
CRP	1.42	4.59 × 10^−8^ ***	1.85	3.60 × 10^−13^ ***	1.18	0.039 *
hs-CRP	1.84	5.97 × 10^−14^ ***	2.24	<2 × 10^−16^ ***	1.30	0.001 ***

OR, odds ratio; Model 1 was unadjusted; Model 2 was adjusted for age, gender, and race. Model 3 was adjusted for age, gender, race, BMI, hyperlipidemia, hypertension, and diabetes mellitus. * *p* < 0.05, ** *p* < 0.01, *** *p* < 0.001.

**Table 2:** Results for subgroup analysis of the association between HUA and NT-pro-BNP, hs-cTnT, LDH, hs-CRP, and CRP.

		Model 1		Model 2		Model 3	
		OR [95% CI]	*p* Value	OR [95% CI]	*p* Value	OR [95% CI]	*p* Value
NT-pro-BNP							
	Q1	Ref	-	Ref	-	Ref	-
	Q2	2.41 [1.16, 4.98]	0.023	1.82 [0.77, 4.27]	0.179	1.75 [0.70, 4.34]	0.237
	Q3	3.22 [1.74, 5.95]	0.001	2.60 [1.25, 5.38]	0.015	2.60 [1.19, 5.65]	0.022
	Q4	5.96 [3.18, 11.15]	<0.001	4.24 [2.11, 8.50]	<0.001	3.97 [1.89, 8.31]	0.001
hs-cTnT							
	Q1	Ref	-	Ref	-	Ref	-
	Q2	1.37 [0.91, 2.08]	0.143	1.00 [0.63, 1.61]	0.981	0.97 [0.58, 1.62]	0.915
	Q3	2.06 [1.43, 2.97]	<0.001	1.63 [1.02, 2.58]	0.047	1.54 [0.93, 2.54]	0.104
	Q4	3.97 [2.65, 5.94]	<0.001	3.40 [2.10, 5.48]	<0.001	3.29 [1.92, 5.63]	<0.001
LDH							
	Q1	Ref	-	Ref	-	Ref	-
	Q2	1.43 [0.83, 2.45]	0.202	1.40 [0.81, 2.43]	0.227	1.40 [0.80, 2.45]	0.240
	Q3	1.75 [1.08, 2.83]	0.024	1.78 [1.10, 2.90]	0.021	1.78 [1.08, 2.91]	0.024
	Q4	2.33 [1.42, 3.81]	0.001	2.20 [1.33, 3.65]	0.003	2.16 [1.28, 3.64]	0.005
CRP							
	Q1	Ref	-	Ref	-	Ref	-
	Q2	0.87 [0.59, 1.30]	0.497	0.79 [0.53, 1.19]	0.263	0.74 [0.48, 1.14]	0.176
	Q3	1.14 [0.77, 1.70]	0.516	1.10 [0.73, 1.66]	0.638	1.00 [0.66, 1.54]	0.969
	Q4	2.15 [1.46, 3.16]	<0.001	1.86 [1.24, 2.79]	0.003	1.65 [1.09, 2.50]	0.020
hs-CRP							
	Q1	Ref	-	Ref	-	Ref	-
	Q2	1.30 [0.85, 1.98]	0.230	1.40 [0.91, 2.13]	0.130	1.33 [0.81, 2.19]	0.267
	Q3	1.07 [0.74, 1.54]	0.739	1.12 [0.76, 1.64]	0.568	0.92 [0.60, 1.41]	0.703
	Q4	2.00 [1.28, 3.11]	0.003	2.09 [1.32, 3.30]	0.003	1.66 [1.02, 2.72]	0.048

OR, odds ratio; CI, confidence interval; Q, quartile; Ref, reference; Model 1 is unadjusted; Model 2 is adjusted for age, gender, and race. Model 3 is adjusted for age, gender, race, BMI, hyperlipidemia, hypertension, and diabetes mellitus.

**Table 3:** Molecular docking binding energy results of UA with cardiac damage markers (BNP, cTnT, LDH, and CRP).

	BNP	cTnT	CRP	LDH
Binding affinity with UA (kcal/mol)	−7.3	−5.2	−6.7	−6.3

## Data Availability

The data used to support the findings of this study are available from the corresponding author upon request.

## Supplementary Materials

The following supporting information can be downloaded at https://www.mdpi.com/article/10.3390/cimb48010040/s1.
