# Supplementary material for: Uric Acid: A New Perspective for Exploring the Pathological Process of Anthracycline-Induced Cardiotoxicity
Source: Curr Issues Mol Biol. 2025 Dec 27;48(1):40. doi: 10.3390/cimb48010040 (PMC12840530; doi:10.3390/cimb48010040)
Supplement: Supplementary file 1 [file cimb-48-00040-s001.zip › cimb-4025342-supplementary.pdf]

Supplementary Table S1. Characteristics of participants included in study, NHANES 1999-2004.

Characteristics	Overall (n = 18024)	Hyperuricemia Yes (n = 1930)	Hyperuricemia No (n = 16094)	*P*-value
Age Mean (SE)	42.24(0.31)	46.47(0.60)	41.67(0.30)	< 0.0001
SUA Mean (SE)	317.10(0.99)	471.05(1.82)	296.22(0.67)	< 0.0001
Height Mean (SE)	168.55(0.12)	173.89(0.27)	167.83(0.13)	< 0.0001
Weight Mean (SE)	78.39(0.28)	92.52(0.74)	76.49(0.28)	< 0.0001
BMI Mean (SE)	27.47(0.10)	30.54(0.23)	27.06(0.09)	< 0.0001
Waist_circumference Mean (SE)	94.37(0.26)	104.83(0.56)	92.97(0.25)	< 0.0001
NT-proBNP Mean (SE)	126.22(3.66)	211.71(17.32)	114.63(4.21)	< 0.0001
Gender (%)				<0.0001
Male	48.50(45.44,51.57)	82.73(80.51,84.96)	43.86(43.04,44.69)	
Female	51.50(48.01,54.98)	17.27(15.04,19.49)	56.14(55.31,56.96)	
Race/Ethnicity (%)				0.01
Non-Hispanic Black	10.37(8.68,12.05)	10.65(8.35,12.95)	10.33(8.45,12.21)	
Non-Hispanic White	71.19(64.00,78.37)	73.54(69.43,77.65)	70.87(67.59,74.14)	
Mexican American	7.69(6.11, 9.27)	5.35(3.85,6.86)	8.01(6.12,9.89)	
Other Race - Including Multi-Racial	4.80(3.72, 5.87)	5.55(3.50,7.61)	4.70(3.76,5.64)	
Other Hispanic	5.96(3.78, 8.14)	4.90(2.39,7.41)	6.10(3.89,8.32)	
Hyperlipidemia				< 0.0001

yes	69.25(64.43,74.06)	83.31(81.33,85.29)	67.34(66.06,68.62)	
no	30.75(28.71,32.79)	16.69(14.71,18.67)	32.66(31.38,33.94)	
Hypertension				< 0.0001
yes	30.92(28.07,33.77)	48.52(45.32,51.71)	28.60(27.21,29.99)	
no	68.90(64.81,72.99)	51.48(48.29,54.68)	71.40(70.01,72.79)	
Diabetes mellitus				< 0.0001
yes	6.35(5.47, 7.23)	9.12(7.58,10.67)	6.10(5.50, 6.70)	
no	91.85(86.21,97.49)	90.88(89.33,92.42)	93.90(93.30,94.50)	
Cardiotoxicity				< 0.0001
yes	4.07(3.49, 4.64)	8.56(7.32,9.81)	3.46(3.05,3.86)	
no	95.93(90.00,101.87)	91.94(90.19,92.68)	96.54(96.14,96.95)	

Mean (SE) was for continuous variables. The percentage (95% confidence interval) was for categorical variables.

NHANES, National Health and Nutrition Examination Survey; SE, standard error; BMI, body mass index; NT-proBNP, N-terminal B-type natriuretic peptide.

Supplementary Table S2. Characteristics of participants included in study, NHANES 1999-2004.

		Hyperuricemia		
Characteristics	Overall (n = 18022)	Yes (n = 1931)	No (n = 16091)	*P*-value
Age	42.25(0.32)	46.49(0.61)	41.68(0.31)	< 0.0001

Mean (SE)				
SUA	317.49(0.96)	471.01(1.82)	296.65(0.62)	< 0.0001
Mean (SE)				
Height	168.56(0.12)	173.89(0.27)	167.84(0.13)	< 0.0001
Mean (SE)				
Weight	78.42(0.29)	92.56(0.73)	76.52(0.28)	< 0.0001
Mean (SE)				
BMI	27.48(0.10)	30.56(0.22)	27.06(0.09)	< 0.0001
Mean (SE)				
Waist_circumference	94.39(0.26)	104.87(0.55)	92.99(0.25)	< 0.0001
Mean (SE)				
hs-cTnT	6.86(0.09)	10.83(0.37)	6.33(0.09)	< 0.0001
Mean (SE)				
Gender (%)				<0.0001
Male	48.55(45.45,51.64)	82.58(80.36,84.81)	43.93(43.09,44.76)	
Female	51.45(47.99,54.92)	17.42(15.19,19.64)	56.07(55.24,56.91)	
Race/Ethnicity (%)				0.004
Non-Hispanic Black	10.35(8.67,12.03)	10.66(8.36,12.97)	10.31(8.44,12.18)	
Non-Hispanic White	71.14(63.96,78.33)	73.36(69.24,77.47)	70.84(67.57,74.12)	
Mexican American	7.70(6.12, 9.27)	5.33(3.82,6.84)	8.02(6.13,9.90)	
Other Race - Including Multi-Racial	4.85(3.76, 5.94)	5.75(3.68,7.83)	4.73(3.78,5.68)	
Other Hispanic	5.96(3.79, 8.13)	4.90(2.39,7.42)	6.10(3.90,8.30)	
Hyperlipidemia				< 0.0001
yes	69.31(64.48,74.15)	83.23(81.27,85.20)	67.42(66.13,68.72)	
no	30.69(28.65,32.72)	16.77(14.80,18.73)	32.58(31.28,33.87)	
Hypertension				< 0.0001
yes	30.88(28.02,33.74)	48.46(45.24,51.68)	28.56(27.16,29.96)	
no	68.94(64.84,73.03)	51.54(48.32,54.76)	71.44(70.04,72.84)	

Diabetes mellitus				< 0.0001
yes	6.35(5.47, 7.23)	9.20(7.64,10.77)	6.08(5.48, 6.69)	
no	91.86(86.22,97.52)	90.80(89.23,92.36)	93.92(93.31,94.52)	
Cardiotoxicity				< 0.0001
yes	7.51(6.65, 8.36)	17.99(16.02,19.97)	6.08(5.55, 6.61)	
no	92.49(86.77,98.22)	82.01(80.03,83.98)	93.92(93.39,94.45)	

Mean (SE) was for continuous variables. The percentage (95% confidence interval) was for categorical variables.

NHANES, National Health and Nutrition Examination Survey; SE, standard error; BMI, body mass index; hs-cTnT, cardiac hs-troponin t.

Supplementary Table S3. Characteristics of participants included in study, NHANES 1999-2016.

		Hyperuricemia		
Characteristics	Overall (n = 53613)	Yes (n = 6147)	No (n = 47466)	*P*-value
Age<br>Mean (SE)	43.07（0.20）	47.30(0.33)	42.51(0.20)	< 0.0001
SUA<br>Mean (SE)	317.67(0.60)	470.41(0.91)	297.46 (0.44)	< 0.0001
Height<br>Mean (SE)	168.22(0.08)	173.12 (0.17)	167.57(0.08)	< 0.0001

Weight Mean (SE)	80.21(0.20)	95.74(0.47)	78.16(0.19)	< 0.0001
BMI Mean (SE)	28.22(0.07)	31.88(0.14)	27.74(0.07)	< 0.0001
Waist_circumference Mean (SE)	96.35(0.18)	107.84(0.37)	94.84(0.18)	< 0.0001
LDH Mean (SE)	139.34(0.42)	145.58(0.72)	138.52(0.42)	< 0.0001
Gender (%)				<0.0001
Male	48.50(46.73,50.28)	81.07(79.78,82.36)	44.19(43.66,44.73)	
Female	51.50(49.59,53.40)	18.93(17.64,20.22)	55.81(55.27,56.34)	
Race/Ethnicity (%)				< 0.0001
Non-Hispanic Black	11.03(9.93,12.13)	11.83(10.24,13.42)	10.93(9.70,12.15)	
Non-Hispanic White	67.01(62.89,71.14)	68.25(65.61,70.89)	66.85(64.72,68.98)	
Mexican American	8.72(7.66, 9.78)	7.10(5.90, 8.30)	8.93(7.76,10.11)	
Other Race - Including Multi-Racial	7.16(6.47, 7.86)	7.85(6.72,8.98)	7.07(6.38,7.76)	
Other Hispanic	6.07(5.13, 7.01)	4.97(3.91,6.03)	6.22(5.24,7.20)	
Hyperlipidemia				< 0.0001
yes	66.67(64.04,69.31)	79.13(77.82,80.44)	65.02(64.18,65.87)	
no	33.33(32.04,34.61)	20.87(19.56,22.18)	34.98(34.13,35.82)	
Hypertension				< 0.0001
yes	31.64(30.25,33.04)	50.52(48.61,52.43)	29.49(28.72,30.26)	
no	67.38(64.89,69.86)	49.48(47.57,51.39)	70.51(69.74,71.28)	
Diabetes mellitus				< 0.0001
yes	8.63(8.17, 9.10)	13.21(12.06,14.35)	8.16(7.77,8.54)	
no	89.97(86.73,93.22)	86.79(85.65,87.94)	91.84(91.46,92.23)	
Cardiotoxicity				< 0.0001
yes	4.16(3.80,4.52)	6.37(5.50,7.25)	3.87(3.53,4.20)	

no	95.84(92.39,99.29)	93.63(92.75,94.50)	96.13(95.80,96.47)	

Mean (SE) was for continuous variables. The percentage (95% confidence interval) was for categorical variables.

NHANES, National Health and Nutrition Examination Survey; SE, standard error; BMI, body mass index; LDH, lactatedehydrogenase.

Supplementary Table S4. Characteristics of participants included in study, NHANES 1999-2010.

		Hyperuricemia		
Characteristics	Overall (n = 39252)	Yes (n = 4445)	No (n = 34807)	*P*-value
Age<br>Mean (SE)	42.27(0.23)	47.00(0.38)	41.62(0.22)	< 0.0001
SUA<br>Mean (SE)	319.03(0.72)	470.72(0.99)	298.36(0.51)	< 0.0001
Height<br>Mean (SE)	168.58(0.08)	173.61(0.16)	167.90(0.09)	< 0.0001
Weight<br>Mean (SE)	79.15(0.22)	94.04(0.48)	77.14(0.22)	< 0.0001
BMI<br>Mean (SE)	27.73(0.07)	31.15(0.15)	27.26(0.07)	< 0.0001
Waist_circumference<br>Mean (SE)	95.06(0.20)	106.14(0.37)	93.59(0.20)	< 0.0001

CRP	0.38(0.01)	0.49(0.02)	0.37(0.01)	< 0.0001
Mean (SE)				
Gender (%)				<0.0001
Male	48.65(46.47,50.83)	81.49(80.20,82.78)	44.17(43.65,44.70)	
Female	51.35(48.96,53.74)	18.51(17.22,19.80)	55.83(55.30,56.35)	
Race/Ethnicity (%)				< 0.0001
Non-Hispanic Black	11.05(9.88,12.23)	11.65(10.01,13.30)	10.97(9.64,12.30)	
Non-Hispanic White	69.79(64.58,75.01)	72.00(69.18,74.82)	69.49(67.00,71.99)	
Mexican American	8.28(7.18, 9.38)	6.05(4.77,7.32)	8.58(7.27,9.90)	
Other Race - Including Multi-Racial	5.57(4.84, 6.31)	6.20(4.90,7.49)	5.49(4.77,6.20)	
Other Hispanic	5.30(4.07, 6.54)	4.10(2.81,5.40)	5.46(4.19,6.74)	
Hyperlipidemia				< 0.0001
yes	68.48(65.06,71.90)	81.85(80.48,83.23)	66.66(65.77,67.54)	
no	31.52(30.15,32.89)	18.15(16.77,19.52)	33.34(32.46,34.23)	
Hypertension				< 0.0001
yes	30.80(28.94,32.67)	49.72(47.68,51.76)	28.29(27.39,29.19)	
no	69.01(66.03,71.09)	50.28(48.24,52.32)	71.71(70.81,72.61)	
Diabetes mellitus				< 0.0001
yes	6.94(6.41, 7.48)	10.86(9.55,12.17)	6.53(6.13, 6.92)	
no	91.49(87.38,95.60)	89.14(87.83,90.45)	93.47(93.08,93.87)	
Cardiotoxicity				< 0.0001
yes	8.86(8.24, 9.48)	11.66(10.53,12.80)	8.48(8.05, 8.90)	
no	91.14(87.11,95.18)	88.34(87.20,89.47)	91.52(91.10,91.95)	

Mean (SE) was for continuous variables. The percentage (95% confidence interval) was for categorical variables.

NHANES, National Health and Nutrition Examination Survey; SE, standard error; BMI, body mass index; CRP, C-reactive protein.

Supplementary Table S5. Characteristics of participants included in study, NHANES 2015-2020.

Characteristics	Overall (n = 21554)	Hyperuricemia<br>Yes (n = 2657)	 No (n = 18897)	*P*-value
Age	44.51(0.33)	47.86(0.52)	44.07(0.34)	< 0.0001
Mean (SE)				
SUA	317.04(0.99)	469.90(1.49)	296.90(0.73)	< 0.0001
Mean (SE)				
Height	167.71(0.14)	172.32(0.33)	167.11(0.14)	< 0.0001
Mean (SE)				
Weight	82.14(0.35)	99.02(0.86)	79.93(0.32)	< 0.0001
Mean (SE)				
BMI	29.08(0.12)	33.25(0.25)	28.54(0.12)	< 0.0001
Mean (SE)				
Waist_circumference	98.62(0.33)	110.75(0.73)	97.03(0.31)	< 0.0001
Mean (SE)				
hs-CRP	3.65(0.08)	5.01(0.21)	3.47(0.08)	< 0.0001
Mean (SE)				
Gender (%)				<0.0001
Male	48.63(45.96,51.29)	80.18(77.95,82.41)	44.47(43.47,45.47)	
Female	51.37(48.59,54.16)	19.82(17.59,22.05)	55.53(54.53,56.53)	
Race/Ethnicity (%)				0.05

Non-Hispanic Black	10.99(9.20,12.77)	12.50(9.75,15.24)	10.79(8.87,12.70)	
Non-Hispanic White	62.17(56.22,68.13)	61.83(57.13,66.53)	62.22(58.77,65.67)	
Mexican American	9.69(7.62,11.75)	8.93(6.59,11.26)	9.79(7.63,11.94)	
Other Race - Including Multi-Racial	9.99(8.72,11.26)	10.65(8.76,12.55)	9.90(8.61,11.19)	
Other Hispanic	7.17(6.14, 8.19)	6.10(4.76,7.43)	7.31(6.18,8.43)	
Hyperlipidemia				< 0.0001
yes	64.11(60.55,67.66)	74.89(72.50,77.28)	62.69(61.19,64.18)	
no	35.89(33.56,38.23)	25.11(22.72,27.50)	37.31(35.82,38.81)	
Hypertension				< 0.0001
yes	32.99(31.03,34.95)	52.71(49.30,56.12)	31.17(29.84,32.51)	
no	64.87(61.11,68.62)	47.29(43.88,50.70)	68.83(67.49,70.16)	
Diabetes mellitus				< 0.0001
yes	11.35(10.60,12.10)	16.89(15.10,18.69)	10.73(10.03,11.43)	
no	87.73(83.02,92.44)	83.11(81.31,84.90)	89.27(88.57,89.97)	
Cardiotoxicity				< 0.0001
yes	33.05(31.23,34.87)	45.69(42.70,48.67)	31.39(30.27,32.50)	
no	66.95(63.13,70.77)	54.31(51.33,57.30)	68.61(67.50,69.73)	

Mean (SE) was for continuous variables. The percentage (95% confidence interval) was for categorical variables.

NHANES, National Health and Nutrition Examination Survey; SE, standard error; BMI, body mass index; hs-CRP, hypersensitive C reactive protein.
